# White matter microstructure is altered in cognitively normal middle-aged *APOE*-ε4 homozygotes

**DOI:** 10.1186/s13195-018-0375-x

**Published:** 2018-05-24

**Authors:** Grégory Operto, Raffaele Cacciaglia, Oriol Grau-Rivera, Carles Falcon, Anna Brugulat-Serrat, Pablo Ródenas, Rubén Ramos, Sebastián Morán, Manel Esteller, Nuria Bargalló, José Luis Molinuevo, Juan Domingo Gispert, for the ALFA Study, Jordi Camí, Gemma Salvadó, Stavros Skouras, Gonzalo Sánchez, Carolina Minguillón, Karine Fauria, Nina Gramunt, Marc Suárez-Calvet, Albina Polo, Cristina Mustata, Laia Tenas, Paula Marne, Xavi Gotsens, Tania Menchón, Anna Soteras, Laura Hernandez, Ruth Dominguez, Sandra Prades, Gema Huesa, Marc Vilanova, Sabrina Segundo, Jordi Huguet

**Affiliations:** 1 Barcelonaβeta Brain Research Center, Pasqual Maragall Foundation, C/ Wellington, 30, 08005 Barcelona, Spain; 2 Centro de Investigación Biomédica en Red de Bioingeniería, Biomateriales y Nanomedicina (CIBER-BBN), Madrid, Spain; 3 Barcelona Supercomputing Center, Barcelona, Catalonia Spain; 4 Cancer Epigenetics and Biology Program (PEBC), Bellvitge Biomedical Research Institute (IDIBELL), L’Hospitalet, Barcelona, Catalonia Spain; 5 Departament de Ciències Fisiològiques II, Escola de Medicina, Universitat de Barcelona, Barcelona, Catalonia Spain; 6 Institució Catalana de Recerca i Estudis Avançats (ICREA), Barcelona, Catalonia Spain; 7 Institut d’Investigacions Biomèdiques August Pi i Sunyer (IDIBAPS), Barcelona, Catalonia Spain; 8 Centre Mèdic Diagnòstic Alomar, Barcelona, Spain; 9 CIBER Fragilidad y Envejecimiento Saludable (CIBERFES), Madrid, Spain

**Keywords:** Diffusion tensor imaging, Apolipoprotein E, White matter integrity, Aging, Cognitively normal subjects

## Abstract

**Background:**

The ε4 allele of the apolipoprotein E gene (*APOE*-ε4) is the strongest genetic factor for late-onset Alzheimer’s disease. During middle age, cognitively healthy *APOE*-ε4 carriers already show several brain alterations that resemble those of Alzheimer's disease (AD), but to a subtler degree. These include microstructural white matter (WM) changes that have been proposed as one of the earliest structural events in the AD cascade. However, previous studies have focused mainly on comparison of *APOE*-ε4 carriers vs noncarriers. Therefore, the extent and magnitude of the brain alterations in healthy ε4 homozygotes, who are the individuals at highest risk, remain to be characterized in detail.

**Methods:**

We examined mean, axial, and radial water diffusivity (MD, AxD, and RD, respectively) and fractional anisotropy in the WM as measured by diffusion-weighted imaging in 532 cognitively healthy middle-aged participants from the ALFA study (ALzheimer and FAmilies) cohort, a single-site population-based study enriched for AD risk (68 *APOE*-ε4 homozygotes, 207 heterozygotes, and 257 noncarriers). We examined the impact of age and *APOE* genotype on these parameters using tract-based spatial statistics.

**Results:**

Healthy *APOE*-ε4 homozygotes display increased WM diffusivity in regions known to be affected by AD. The effects in AxD were much smaller than in RD, suggesting a disruption of the myelin sheath rather than pure axonal damage.

**Conclusions:**

These findings could be interpreted as the result of the reduced capacity of the ε4 isoform of the APOE protein to keep cholesterol homeostasis in the brain. Because cerebral lipid metabolism is strongly related to the pathogenesis of AD, our results shed light on the possible mechanisms through which the *APOE*-ε4 genotype is associated with an increased risk of AD.

**Electronic supplementary material:**

The online version of this article (10.1186/s13195-018-0375-x) contains supplementary material, which is available to authorized users.

## Background

The ε4 allele of the apolipoprotein E gene (*APOE*-ε4) is the strongest genetic factor for late-onset Alzheimer’s disease. Compared with those individuals with an *APOE* ε3/ε3 genotype, white individuals with one copy of the ε4 allele show an increased lifetime risk of developing Alzheimer's disease (AD) (ε2/ε4, OR 2.6; ε3/ε4, OR 3.2). The risk is much higher for carriers of two copies (ε4/ε4, OR 14.9) [1]. The main roles of the ApoE protein, encoded by the *APOE* gene, include lipid transport and clearance of amyloid deposition. However, the ε4 isoform of the ApoE protein shows an impaired capacity to perform these functions compared with the other isoforms [2]. Such impaired function may underlie the observed effects of *APOE-*ε4 on the brain throughout the lifespan. In particular, *APOE-*ε4 has been related to earlier and increased amyloid-β deposition, one of the neuropathological hallmarks of AD [3, 4]. However, the effects on brain morphology have been reported to be subtler [5]. Most of the studies so far have stratified individuals in only two levels of risk (*APOE-*ε4 carriers vs noncarriers). However, *APOE-*ε4 homozygotes, who completely lack expression of the most efficient isoform of the ApoE protein, are an interesting population to study to gain a better understanding of the mechanisms through which *APOE* genotype modulates the risk of AD. Given the essential implication of ApoE in the transport of cholesterol, the main component of the myelin sheath, it is conceivable that alterations in white matter (WM) microstructure may be one of these mechanisms.

The last decade has seen increasing interest in the study of brain microstructure measured using diffusion magnetic resonance imaging (dMRI). Water molecules are locally influenced by existing axon fibers [6], and their movements properties can be described by a set of measures generally including fractional anisotropy (FA), mean diffusivity (MD), axial diffusivity (AxD), and radial diffusivity (RD). Variations in these parameters can capture microstructural changes such as axonal loss, inflammation, Wallerian degeneration [7], demyelination, or fiber damage [8], and their alteration is likely to hinder transfer of information across networks, eventually leading to cognitive impairment [9–13].

There is a growing body of evidence supporting the association between *APOE*-ε4 status and WM integrity in cognitively normal subjects as measured using diffusion (or relaxation) parameters. The nature of this association, however, is still under debate. WM alterations have been detected in individuals at genetic risk of AD [14–18]. Persson et al. and Honea et al. [15, 17] reported decreased anisotropy in ε4 carriers compared with noncarriers. Heise et al. [14] compared two groups comprising young (aged 20–35 years) and old (aged 50–78 years) participants (*N* = 73) and found a general reduction of FA and a general increase in MD in ε4 carriers. Westlye et al. [18] observed widespread increases in MD and RD in carriers of the ε4/ε3 alleles compared with ε3/ε3 in 203 volunteers aged 21.1–69.9 years. Recently, Cavedo et al. [19] studied 74 participants (mean age 67.85 years) and found a significant reduction of FA and increase in RD in ε4 carriers vs noncarriers.

Some researchers have described a genotypic effect that remains stable throughout life, with ε4 carriers showing local increased diffusivity and lower FA in an age-independent manner [14–18]. In contrast, other studies have suggested that *APOE* instead impacts the trajectory of age-related changes [12, 20], with ε4 carriers showing accelerated diffusion changes across the older adult age range. Assessing interaction between age and genotype is challenging without a longitudinal design, as reflected by the inconsistency in the findings from these cross-sectional datasets [21]. Regarding these previous studies, it is worth noting the large existing variability in the age range of the participants, the ROIs, the sample size, the number of ε4 carriers, or the employed methodology.

WM alterations have also been found in patients with AD [22] and patients with mild cognitive impairment [23, 24]. Interestingly, it has been proposed that APOE may play a role in modulating the focality of these alterations [25]. Such microstructural effects on WM add to the well-known effect of APOE on gray matter (GM) morphology across the AD continuum [26, 27], driving the neuroanatomical expression of the most common variant AD phenotypes [28], or even in cognitively healthy middle-aged individuals [29]. However, only a few studies have described WM differences in the preclinical state of the disease (i.e., cognitively healthy individuals with altered amyloid biomarkers) by addressing the hypothesis that the preclinical state of AD is distinct from normal aging [30–32]. Their final conclusions unanimously identified dMRI metrics as promising markers of early degeneration, potentially predating changes at a macrostructural level. A summary of the studies examining the APOE polymorphisms ε2/ε3/ε4 and WM integrity using dMRI are listed in Table 1 [33].

**Table 1:** Studies examining the apolipoprotein E polymorphisms ε2/ε3/ε4 and white matter integrity using diffusion magnetic resonance imaging

Reference (year)	Methods	Measures	Sample size	Age (years)	Genotype groups	Homozygotes (no.)	Results
Nierenberg et al. (2005) [16]	ROI	FA, AxD, RD	29	67.1 (6.5)	14 ε4 carriers 15 ε4 noncarriers	2	ε4 carriers: ↓ FA and ↑ RD in L parahippocampal gyrus (*p* = 0.015)
Persson et al. (2006) [17]	ROI, SPM-VBM	FA	60	66.3 (7.7)	Two analyses: 10 ε4/ε4–10 ε3/ε4–10 ε3/ε3 30 ε4 carriers, 30 ε3/ε3	10	↓ FA in ε4 carriers: Posterior corpus callosum and fronto-occipital fasciculus No evidence of dose-dependent effect, but not enough data
Honea et al. (2009) [15]	TBSS	FA	53	73.4 (6.3) > 60	39 ε3/ε3 12 ε3/ε4 2 ε4/ε4	2	↓ FA in ε4 carriers: L parahippocampal gyrus (*p* < 0.001 uncorrected)
Smith et al. (2010) [51]	TBSS	FA	65	62.9 (1.3)	42 ε4 carriers 23 ε4 noncarriers	n/a	↓ FA in LOAD risk group in many regions (e.g., bilateral inferior fronto-occipital fasciculus, cingulum bundle, splenium) (*p* < 0.01)
Gold et al. (2010) [42]	TBSS	MD, FA, RD, AxD	57	58.9 (5.8)	37 ε4 carriers with FH 20 ε4 noncarriers without FH	n/a	Significant for LOAD risk group only: ↓ FA ↑ RD: inferior longitudinal fasciculus, inferior fronto-occipital fasciculus/uncinate fasciculus (*p* < 0.001) ↓ FA: Fornix, ↑ MD: Genu and R inferior fronto-occipital fasciculus/inferior longitudinal fasciculus, ↓ AD: Cingulum
Bendlin et al. (2010) [30]	SPM-VBM	FA, MD	136	69.2 (10.2)	56 ε4 carriers 80 ε4 noncarriers	n/a	No significant interactions between genotype and age were observed ε4 allele: not significant Family history LOAD + ε4: ↓ FA in multiple brain regions
Heise et al. (2011) [14]	TBSS	MD, FA, RD, AxD	73	(1) Young 28.6 (4.2) (2) Older: 64.9 (7.19)	17 ε4 carriers, 17 ε4 noncarriers (younger) 16 ε4 carriers, 21 ε4 noncarriers (older)	n/a	ε4 carriers: ↑ MD (older) and ↓ FA (younger) in many regions (e.g., cingulum, corpus callosum) (*p* < 0.05)
Ryan et al. (2011) [12]	ROI	FA, ADC	126	CN (52–92)	88 ε4 noncarriers 32 ε4 heterozygotes 6 ε4/ε4	6	ε4 carriers: ↑ ADC with ↑ age in all regions (*p* < 0.0001) ↓ FA with ↑ age: Frontal, and temporal WM, genu (*p* < 0.05)
Westlye et al. (2012) [18]	TBSS	MD, FA, RD, AxD	203	47.6 (14.9) 21.1–69.9	30 ε2/ε3 113 ε3/ε3 60 ε3/ε4	0	ε4 carriers: widespread increases in MD and RD no interaction between age and genotype no significant differences between ε2/ε3 and ε3/ε4
Adluru et al. (2014) [20]	ROI	MD, FA, RD, AxD	343	61.03 (6.72) 47–76	14 ε4/ε4 109 ε4 heterozygotes 220 ε4 noncarriers	14	Subjects with FH: higher AxD in ε4 carriers, lower AxD in ε4 non-carriers, both in the uncinate fasciculus ε4 carriers: higher MD in the SLF (older) and in the portion of the cingulum bundle running adjacent to the cingulate cortex, also higher RD in the genu
Kljajevic et al. (2014) [62]	ROI	FA, MD	56	67.7 (5.9)	28 ε4 carriers, 28 ε4 noncarriers	n/a	ε4 carriers: higher MD in healthy controls but not in AD (*p* < 0.001, uncorrected)
Lyall et al. (2014) [63]	ROI	FA	645	72.70 (0.74)	2 ε2/ε2 77 ε2/ε3 14 ε2/ε4 376 ε3/ε3 160 ε3/ε4 13 ε4/ε4	13	ε4 carriers: lower FA in right ventral cingulum and left inferior longitudinal fasciculus
Laukka et al. (2015) [64]	TBSS	FA, MD	89	81.41 (3.01)	23 ε4 carriers, 66 ε4 noncarriers	n/a	ε4 carriers: lower FA in forceps major and higher MD in corticospinal tract
Cavedo et al. (2017) [19]	TBSS	MD, FA, RD, AxD	74	68.95 (6.85)	31 ε4 carriers, 43 ε4 noncarriers	n/a	ε4 carriers: lower FA and higher RD in the cingulum, corpus callosum, inferior fronto-occipital and in the inferior longitudinal fasciculi, also higher MD in the genu, right internal capsule, superior longitudinal fasciculus and corona radiata.

*Abbreviations*: *FH* Family history, *LOAD* Late-onset Alzheimer’s disease, *VBM* Voxel-based morphometry, *ADC* Apparent diffusion coefficient

Adapted from [33]

The purpose of the present study was to add to the existing literature by evaluating genotype-related differences in WM integrity as captured by diffusion parameters in a cohort of cognitively normal middle-aged individuals at three levels of AD risk (noncarriers, ε4 heterozygotes, and ε4 homozygotes). We hypothesized that subjects at higher risk of developing AD would show more pronounced age-related changes and therefore more negatively affected microstructure. Such changes would manifest mainly as higher diffusivity in *APOE-*ε4 carriers, especially among homozygotes. FA is not expected to be significantly altered in this population comprising middle-aged cognitively healthy participants. We hypothesized that these changes may appear in regions involved in AD pathogenesis, in particular along bilateral long associative tracts. The present cohort also allowed us to recruit an unprecedented number of individuals homozygous for the ε4 risk allele for a single-site cohort to better understand this allele’s neurobiological impact on brain microstructure. We analyzed DW parametric maps—namely FA, MD, RD, and AxD—using a skeleton-based approach focused on WM tracts. We assessed effects of *APOE*-ε4 load, status, age, and sex. Age by genotype interaction was also tested. Given some previous reports in the literature showing associations between cognitive functions and the integrity of the WM, the effect of educational attainment was also assessed on every parameter.

## Methods

### Study participants

The recruitment for the study consisted of two steps. First, 2743 cognitively healthy volunteers aged between 45 and 74 years were enrolled in the ALFA study (ALzheimer and FAmilies), a large cohort program aimed at identifying neuroimaging biomarkers of preclinical AD in the general population [34]. Exclusion criteria included performance below established cutoffs for a number of cognitive tests as well as the presence of any psychiatric or any other clinically significant condition [34]. Second, after *APOE* genotyping, all participants homozygous for the ε4 allele and all carriers of the ε2 allele were invited to undergo magnetic resonance imaging (MRI), along with ε4-heterozygous and noncarriers matched for age and sex. This sampling strategy resulted in 576 study participants, 44 of whom had to be excluded because of either MRI incidental findings or poor image quality, resulting in a final sample size of 532. Demographic characteristics of the participants are summarized in Table 2.

**Table 2:** Sample characteristics

	Total sample (*N* = 532)		NC (*n* = 257)		HE (*n* = 207)		HO (*n* = 68)		Inferential statistics
	Mean	SD	Mean	SD	Mean	SD	Mean	SD	
Age, years	58.13	7.46	58.52	7.62	58.71	7.41	54.94	6.22	*F* = 7.37; *p* < 0.01
Education, years	13.61	3.54	13.61	3.60	13.59	3.52	13.38	3.48	*F* = 0.19; *p* = 0.83
MMSE score	29.06	1.05	29.00	1.12	29.02	1.11	29.26	0.78	*F* = 1.45; *p* = 0.24
TFR^a^	16.46	5.16	16.32	5.20	16.24	5.09	17.73	5.16	*F* = 2.25; *p* = 0.11
TPR^a^	24.13	4.48	23.83	4.85	24.16	4.19	25.19	3.71	*F* = 2.36; *p* = 0.10
Males/females, *n*	211/321		92/165		94/113		25/43		χ^2^ = 4.79; *p* = 0.09

*Abbreviations: NC* Noncarriers; *HE* ε4-Heterozygous; *HO* ε4-Homozygous; *MMSE* Mini Mental State Examination; *TPR* Total paired recall; *TFR* Total free recall

^a^Full evaluation of cognitive performance was not available for 16 subjects

For the statistical analyses, participants were pooled according to the cumulative number of ε4 alleles, that is, noncarriers as well as ε4-heterozygous and ε4-homozygous individuals. However, homozygous subjects were significantly younger than noncarriers and heterozygotes (Table 1). For this reason, age was included as a covariate in all subsequent analyses. In order to account for potential bias due to these differences, a secondary analysis was also performed using age-matched subgroups. The study was approved by the local ethics committee, and all participants provided written informed consent to participate in the study.

### *APOE* genotyping

Total DNA was obtained from the blood cellular fraction by proteinase K digestion followed by alcohol precipitation. Samples were genotyped for two single-nucleotide polymorphisms (rs429358 and rs7412) determining the possible *APOE* isoforms ε1, rs429358 (C) + rs7412 (T); ε2, rs429358 (T) + rs7412 (T); ε3, rs429358 (T) + rs7412 (C); and ε4, rs429358 (C) + rs7412 (C). Of the 532 participants, 162 were ε3/ε4 carriers, 153 were homozygous for the ε3 allele, 104 were ε2/ε3 carriers, 68 were homozygous for the ε4 allele, and 45 were ε2/ε4. The allele frequencies were in Hardy-Weinberg equilibrium.

### Image data acquisition

All brain MRI data were acquired on a single standard 3-T scanner (GE Discovery MR750w; GE Healthcare Life Sciences, Marlborough, MA, USA). The dMRI protocol employed a spin-echo echo-planar imaging sequence with one T2-weighted baseline (b = 0 s·mm^− 2^), 64 b = 1000 s·mm^− 2^ DW volumes acquired with 64 distinct diffusion-encoding directions. The field of view was 256 × 256 mm, and the imaging matrix was 128 × 128 with 56 slices and slice thickness 2 mm, giving 2-mm isotropic voxels.

### Image processing

DW images were first corrected for eddy current distortions and then denoised with the overcomplete local PCA method described by Manjón et al. [35]. Data analysis was then performed using tools from the FMRIB Software Library software suite [36] (http://www.fmrib.ox.ac.uk/fsl). FA, MD, AxD, and RD maps were generated using DTIFit, which is part of FSL that fits a diffusion tensor model at each voxel. The FA output images were used as input for tract-based spatial statistics (TBSS) [37]. All subjects’ FA data were coregistered to a common space using FMRIB’s Non-linear Image Registration Tool. A mean FA image was generated, and a mean FA skeleton was created using TBSS, which represents the centers of all tracts common to the group. The mean skeleton was thresholded and binarized at FA > 0.2. Each subject’s aligned FA data were then projected onto this skeleton, and the resulting data were fed into voxelwise general linear model cross-subject statistics. Similar warping and analyses were used for each parametric map (FA, MD, AxD, and RD) using the FA skeleton as a reference template.

### Statistical analysis

Group-related differences were assessed using a voxel-by-voxel permutation nonparametric test (5000 permutations) with threshold-free cluster enhancement, performed using the Randomise tool available in FSL [38]. All results are shown at the *p* < 0.05 significance level corrected for multiple comparisons across space. Correction for multiple testing was applied using the default familywise error rate control with threshold-free cluster enhancement as implemented in Randomise and as described elsewhere [38, 39]. For each of the four diffusion parameters (MD, FA, RD, and AxD), we performed a voxelwise multiple linear regression analysis using two main models, the first one to measure the effect of the *APOE* genotype on diffusion parameters and the second to model the interaction with age on top of the main effect of genotype. We partitioned genetic variance by including three dummy regressors coding for the number of ε4 alleles carried. The first model included age and sex as confounding variables. The second one included only sex, because age-by-genotype interaction was the effect of interest. Separate *t* test contrast weights were specified to compare MD, FA, RD, and AxD maps to assess the different components of the effects of the *APOE-*ε4 genotype among dominance (ε4 carriers vs noncarriers), recessivity (homozygotes vs others), and additivity (correlation with the number of ε4 alleles carried) in both directions.

### Supplementary analyses

These analyses were completed by performing a set of supplementary experiments intended to control for potential confounding effects in our dataset. In order to control for the observed difference in age between *APOE* ε4 homozygotes and other subjects, we performed an additional analysis using exactly the same protocol as described in the two preceding subsections, but we restricted it to an age-matched subsample. Also, to discard the potential influence of the ε2 allele in the group of ε4 noncarriers, ε2 carriers were left out of this subsample. The resulting dataset amounted to 65 noncarriers ε3/ε3, 65 *APOE* ε4 heterozygotes ε3/ε4, and 65 *APOE* ε4 homozygotes. Results of this analysis are presented in Additional file 1: Appendix A.

An additional voxel-wise whole-brain analysis was performed using the Statistical Parametric Mapping (SPM) suite to explore the possible extensions of our results releasing the skeleton-based spatial constraint and using the exact same statistical models. Methods and results are detailed in Additional file 1: Appendix B. To rule out any potential local effects of white matter hyperintensities (WMH), a complementary analysis was performed, masking out any voxel segmented as WMH in any subject of the studied sample. WMH masks were segmented from fluid-attenuated inversion recovery images of the same individuals using a method described previously [40]. Global effects of WMH load were also assessed by introducing global WMH volumes and Fazekas scores as confounders in the statistical models. Finally, the level of educational attainment was assessed as an additional potential confounder.

## Results

### Effect of *APOE* genotype

**Fig. 1:**
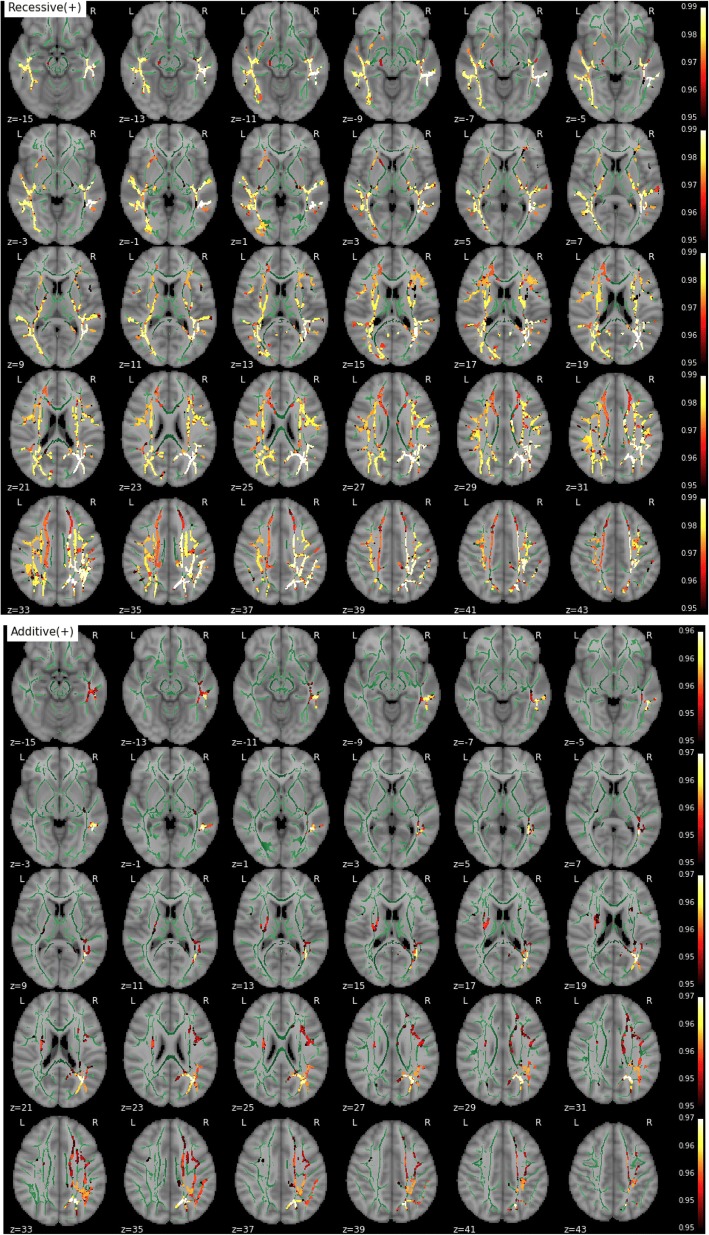
Effect of *APOE* on mean diffusivity (MD) (from top to bottom: recessive and additive components). No dominant component was observed. Only contrast maps associated with higher MD in ε4 carriers showed significant voxels. The white matter skeleton is shown in *green*. Suprathreshold clusters are presented in colors from *dark red* to *white* (1 − *p* > 0.95, familywise error rate- and threshold-free cluster enhancement-corrected)

We found significant main effects of *APOE-*ε4 on MD, RD, and AxD, whereas no significant effect of the risk variant on FA was detected. Figure 1a shows the TBSS map for MD resulting from the recessive contrast, where *APOE-*ε4 homozygotes displayed increased parametric value compared with both noncarriers and heterozygotes in extended bilateral regions of the skeletonized WM, including, in decreasing order of cluster size, corona radiata, superior longitudinal fasciculus (SLF), inferior longitudinal fasciculus (ILF), inferior fronto-occipital fasciculus (IFOF), and corticospinal tract. Interestingly, we also found a significant additive effect of *APOE-*ε4 on MD, which was more pronounced in the right hemisphere along the SLF and ILF and the IFOF (Fig. 1b). The contrast assessing the dominance effect (*APOE*-ε4 noncarriers vs others) did not yield significant group differences.

**Fig. 2:**
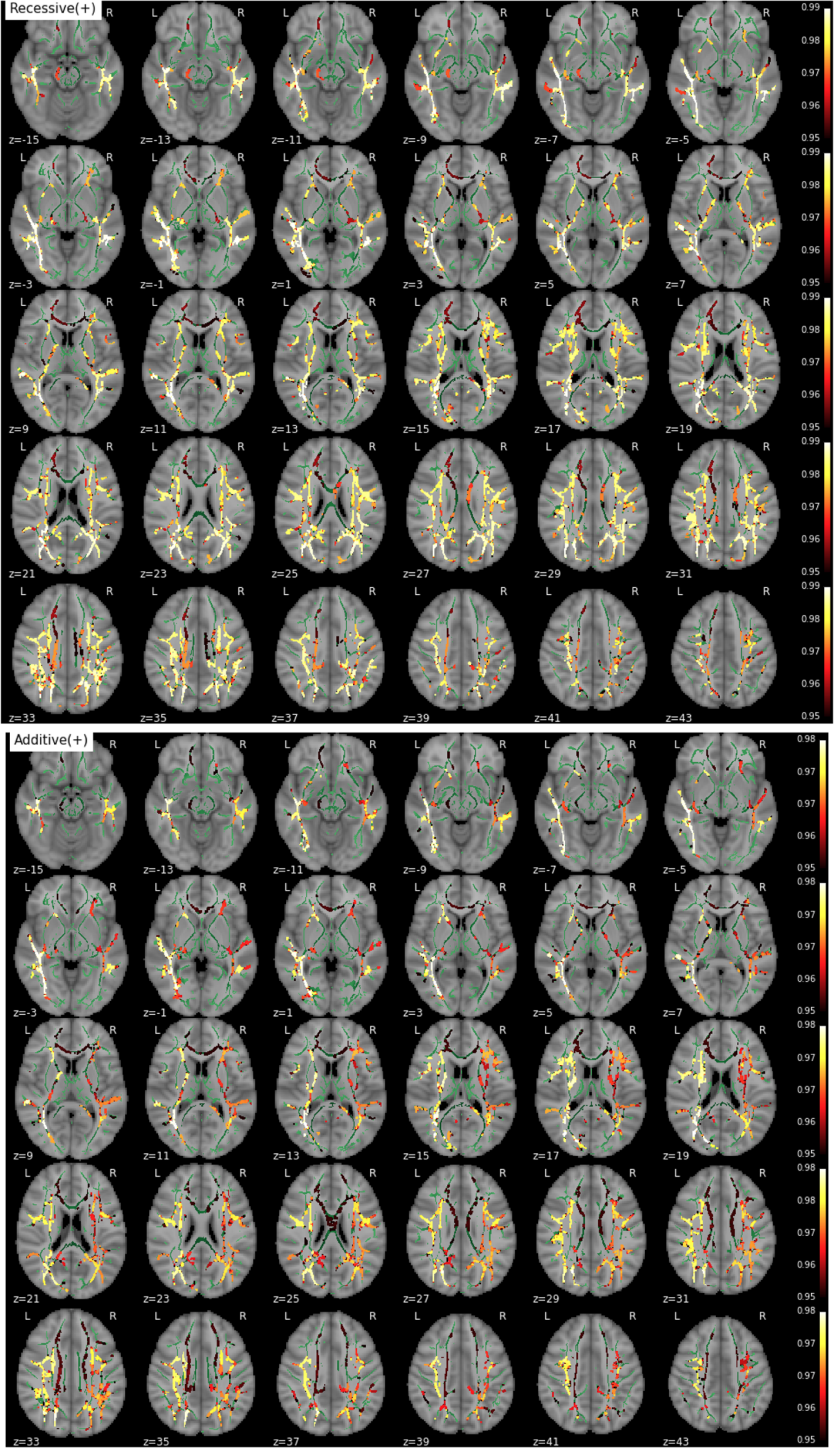
Effect of *APOE* on radial diffusivity (RD) (from top to bottom: recessive and additive components). No dominant component was observed. Only contrast maps associated with higher RD in ε4 carriers showed significant voxels. The white matter skeleton is shown in *green*. Suprathreshold clusters are presented in colors from *dark red* to *white* (1 − *p* > 0.95, familywise error rate- and threshold-free cluster enhancement-corrected)

**Fig. 3:**
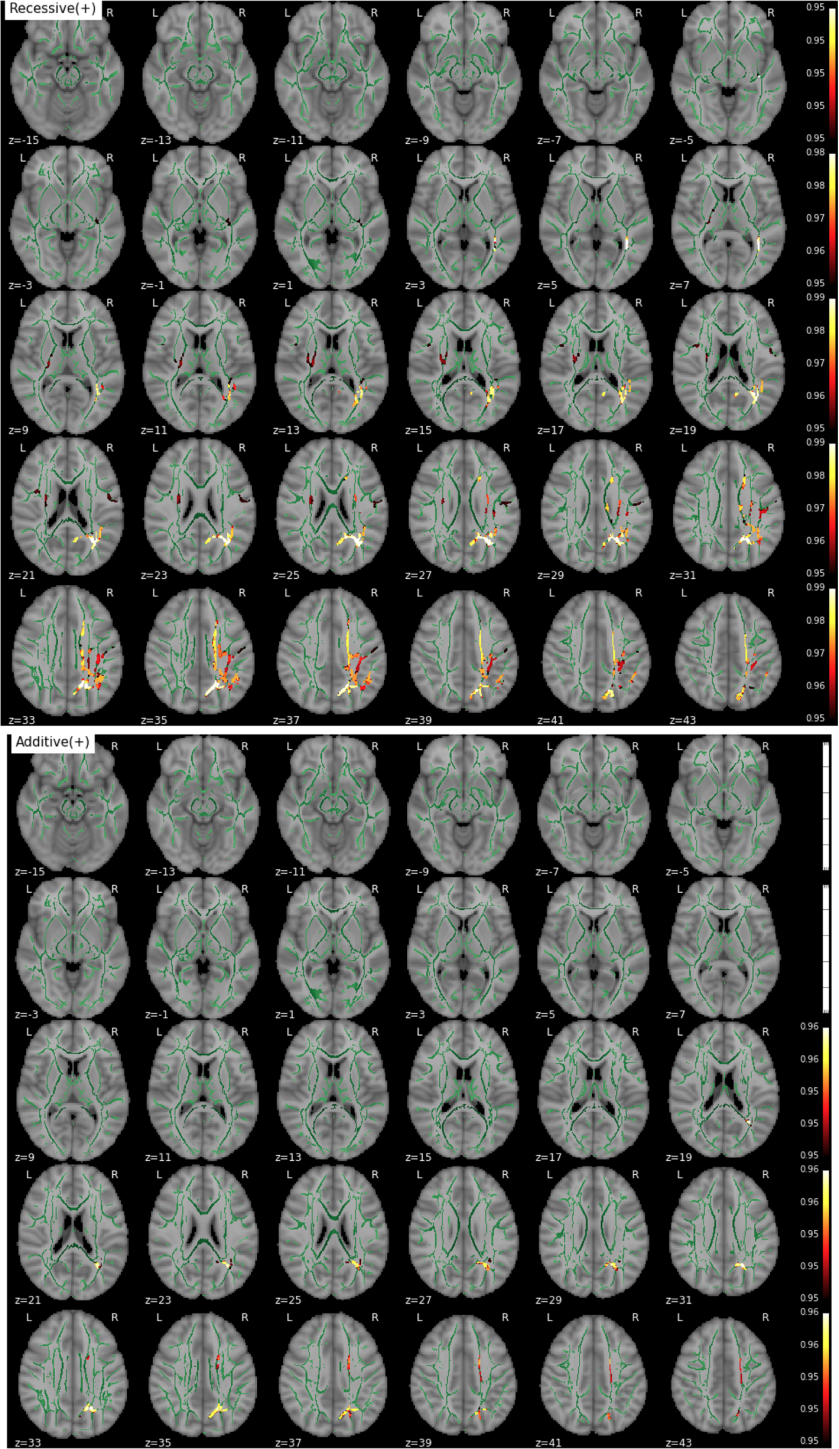
Effect of *APOE* on axial diffusivity (AxD) (from top to bottom: recessive and additive components). No dominant component was observed. Only contrast maps associated with higher AxD in ε4 carriers showed significant voxels. The white matter skeleton is shown in *green*. Suprathreshold clusters are presented in colors from *dark red* to *white* (1 − *p* > 0.95, familywise error rate- and threshold-free cluster enhancement-corrected)

**Fig. 4:**
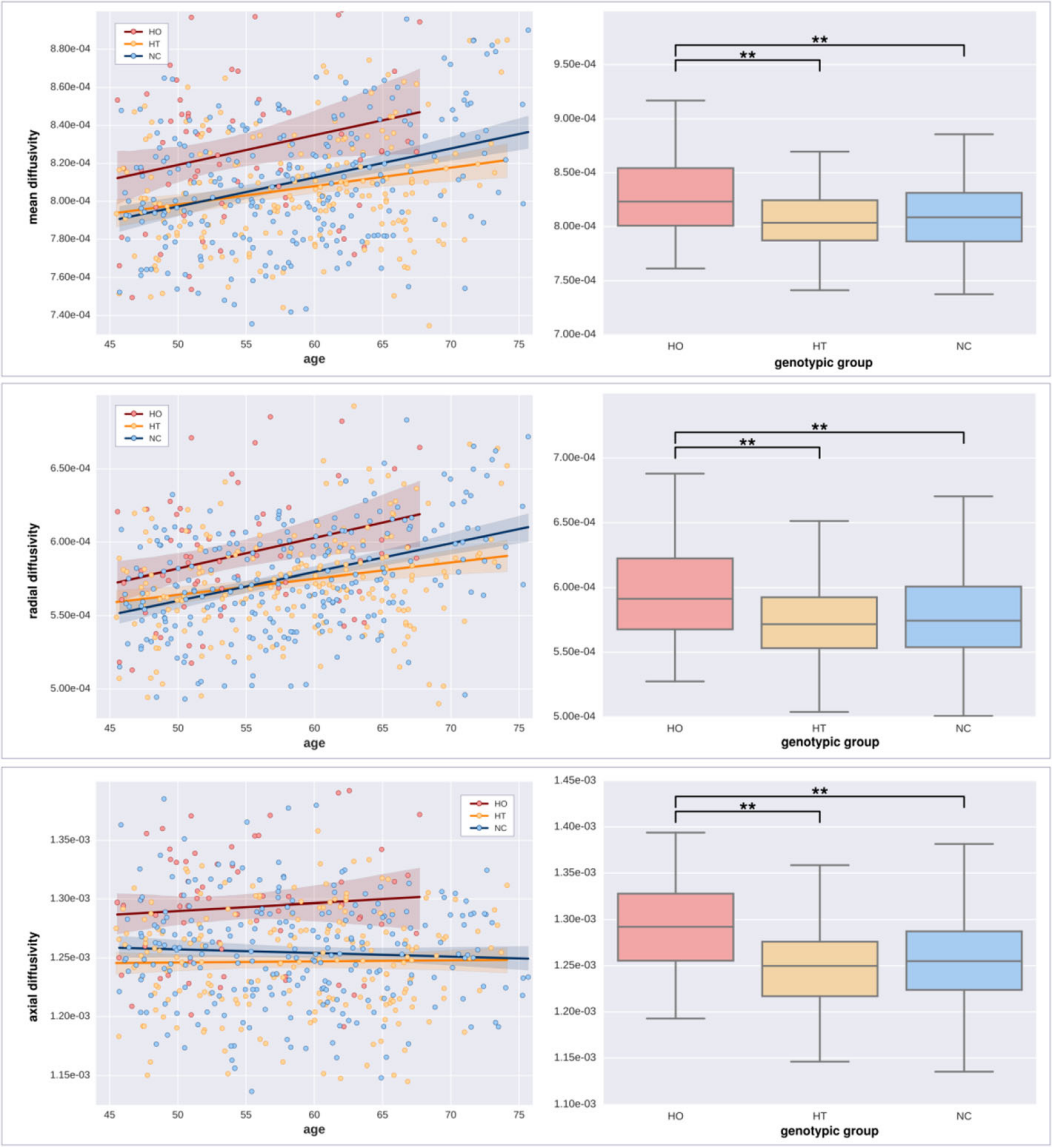
Effect of aging and *APOE* genotype on diffusion parameters (in seconds per mm^2^) on significant voxels in the recessive contrast. *Left*: Scatterplots of regional diffusivity across subjects (MD, RD, and AxD from top to bottom) regressed by age (*solid lines*). *Right*: Box plots based on genotype groups (ε4 homozygotes [HO], ε4 heterozygotes [HT], and noncarriers [NC] from left to right). Asterisks depict significance after a post hoc *t* test (*p* < 0.001, uncorrected)

**Fig. 5:**
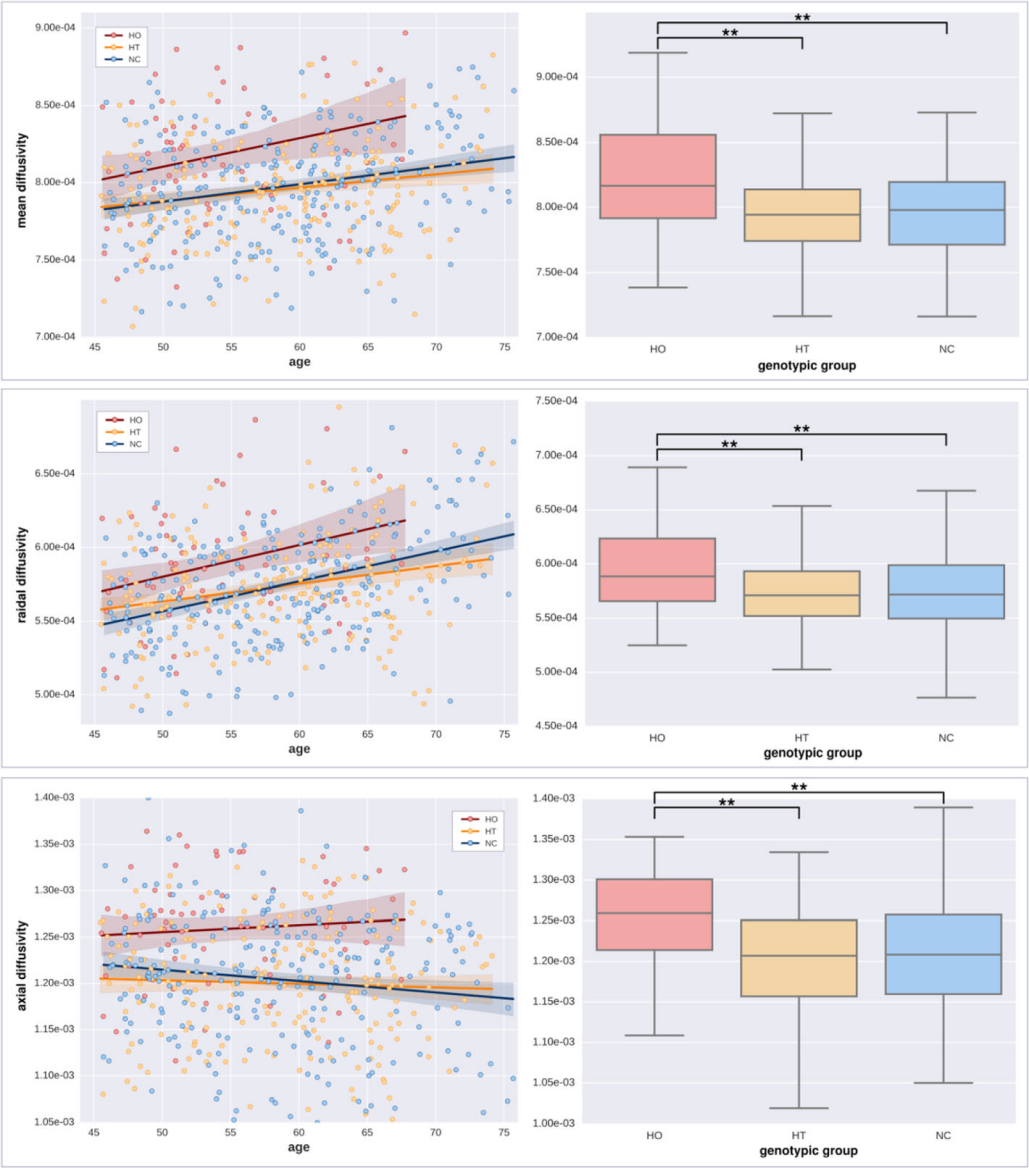
Effect of aging and *APOE* genotype on diffusion parameters (in seconds per mm^2^) on significant voxels in the additive contrast. *Left*: Scatterplots of regional diffusivity across subjects (MD, RD, and AxD from top to bottom) regressed by age (*solid lines*). *Right*: Box plots based on genotype groups (ε4 homozygotes [HO], ε4 heterozygotes [HT], and noncarriers [NC] from left to right). Asterisks depict significance after a post hoc *t* test (*p* < 0.001, uncorrected)

Consistent with the results for MD, we found significantly increased RD and AxD in *APOE*-ε4 homozygotes in both the recessive (homozygotes vs others) and additive contrasts (positive association with the number of ε4 alleles), whereas no significant effects were detected in the dominant contrast (noncarriers vs others). RD increases followed a pattern very close to that of MD (Fig. 2). Regions showing a recessive effect included bilateral SLF, ILF, IFOF, and forceps minor, with 83% of suprathreshold voxels shared between RD and MD (Fig. 2a). Clusters demonstrating an additive effect shared again over 88% of suprathreshold voxels with MD (Fig. 2b). Regions showing AxD increases in *APOE*-ε4 were less extended and stronger in the right hemisphere (Fig. 3). Clusters suggesting a recessive effect appeared in regions such as right SLF, right IFOF, and right corticospinal tract in decreasing order of spatial extent, sharing 15% of significant voxels with MD (Fig. 3a). Finally, an additive effect was found in regions similar to those in the recessive map. However, these regions were much more reduced in spatial extent with an 11% of overlap with significant voxels observed with MD (Fig. 3b). Values derived from these significant clusters are plotted in Figs. 4 and 5 with respect to age and genotype group for MD, RD, and AxD.

In all the analyses performed, *APOE*-ε4 homozygotes never significantly showed lower levels in MD, RD, and AxD, and they did not show higher levels in FA. Levels in FA did not significantly differ between groups in our dataset in either a recessive or a dominance effect. No significant interaction between age and genotype was observed for any of the analyses performed. Table 3 summarizes the tracts for which APOE status showed a significant impact on one or more diffusion metrics.

**Table 3:** Tracts for which apolipoprotein E status showed a significant effect on diffusion metrics

	MD							AxD							RD							FA
	Recessive			Additive			Dom.	Recessive			Additive			Dom.	Recessive			Additive			Dom.	
Name of tract	t	p	size	t	p	size		t	p	size	t	p	size		t	p	size	t	p	size		
Anterior thalamic radiation L	2,57	0,02	11,45	n.s.			n.s.	n.s.			n.s.			n.s.	2,74	0,02	21,78	2,94	0,02	30,47	n.s.	n.s.
Anterior thalamic radiation R	3,11	0,02	7,08	3,25	0,05	0,33		n.s.			n.s.				3,50	0,02	13,02	3,26	0,03	11,89		
Corticospinal tract L	3,15	0,02	43,29	2,70	0,05	2,92		3,44	0,04	7,28	n.s.				3,98	0,02	48,84	3,48	0,03	32,33		
Corticospinal tract R	3,09	0,01	52,32	3,00	0,05	12,68		3,05	0,02	37,65	2,87	0,05	7,81		3,11	0,02	36,13	2,91	0,03	15,57		
Forceps major	3,82	0,01	38,51	3,69	0,04	8,59		4,52	0,02	14,39	n.s.				3,69	0,01	39,94	3,50	0,02	37,25		
Forceps minor	3,88	0,03	5,58	n.s.				n.s.			n.s.				3,56	0,04	40,51	3,06	0,04	38,88		
Inferior fronto-occipital fasciculus L	4,17	0,01	58,59	n.s.				n.s.			n.s.				4,37	0,01	70,08	4,47	0,02	70,04		
Inferior fronto-occipital fasciculus R	4,59	0,01	40,72	4,39	0,04	11,94		3,43	0,02	9,50	n.s.				3,83	0,01	56,33	3,60	0,03	50,39		
Inferior longitudinal fasciculus L	4,57	0,01	72,19	n.s.				n.s.			n.s.				4,41	0,01	82,47	3,95	0,02	78,42		
Inferior longitudinal fasciculus R	3,89	0,01	78,71	3,95	0,04	52,65		n.s.			n.s.				3,43	0,01	85,58	3,60	0,03	84,09		
Superior longitudinal fasciculus (SLF) L	4,18	0,02	63,22	n.s.				3,53	0,05	0,19	n.s.				3,39	0,01	66,94	3,25	0,02	61,33		
SLF (temporal part) L	1,82	0,02	100,00	n.s.				n.s.			n.s.				1,72	0,02	100,00	1,07	0,04	100,00		
Superior longitudinal fasciculus (SLF) R	4,31	0,01	59,52	3,69	0,04	34,73		4,09	0,02	35,60	n.s.				4,50	0,01	69,41	3,82	0,02	55,17		
SLF (temporal part) R	4,88	0,01	81,36	4,07	0,03	71,19		3,52	0,03	18,87	n.s.				4,64	0,01	82,46	4,36	0,02	80,00		
Uncinate fasciculus L	2,67	0,02	52,00	n.s.				n.s.			n.s.				2,83	0,02	68,60	2,39	0,02	60,49		
Uncinate fasciculus R	n.s.			n.s.				n.s.			n.s.				n.s.			n.s.				
Fornix (cres) R	2,59	0,02	11,23	n.s.				n.s.			n.s.				2,08	0,03	14,02	2,07	0,03	29,17		
Fornix (cres) L	4,55	0,01	53,71	3,91	0,03	30,75		4,09	0,01	31,81	n.s.				4,57	0,01	64,15	3,70	0,02	50,16		
Anterior corona radiata R	3,36	0,02	38,36	n.s.				n.s.			n.s.				3,14	0,02	51,45	2,97	0,02	48,93		
Anterior corona radiata L	3,58	0,01	74,94	3,24	0,04	29,71		3,52	0,02	47,64	3,55	0,04	15,65		3,70	0,02	54,34	3,51	0,03	51,81		
Superior corona radiata R	3,73	0,02	78,01	3,83	0,04	18,51		3,35	0,04	10,49	n.s.				3,29	0,02	78,25	3,15	0,02	78,28		
Superior corona radiata L	3,58	0,01	68,31	3,18	0,03	40,09		3,76	0,01	42,37	3,33	0,04	11,17		3,30	0,01	76,92	3,20	0,03	65,98		
Posterior corona radiata R	3,42	0,01	43,82	n.s.				n.s.			n.s.				3,36	0,01	53,02	2,62	0,02	37,54		
Posterior corona radiata L	3,87	0,01	64,26	3,60	0,03	36,48		3,65	0,02	54,46	n.s.				3,74	0,01	66,00	3,37	0,03	59,06		

*Abbreviations: MD* Mean diffusivity, *AxD* Axial diffusivity, *RD* Radial diffusivity

*Note*: “Recessive contrast”: APOE-ε4 homozygotes > others; “additive contrast”: positive association with the number of carried APOE-ε4 alleles; dom. “dominant contrast”: APOE-ε4 carriers > APOE-ε4 noncarriers

ROIs are taken using the intersection between the skeleton produced by TBSS and the John Hopkins University tract-based white matter atlas [65]. The table also reports the most significant t and *p* values (corrected using FWE-TFCE) and the relative size (as % of total tract size) of the significant clusters

### Effect of other factors

**Fig. 6:**
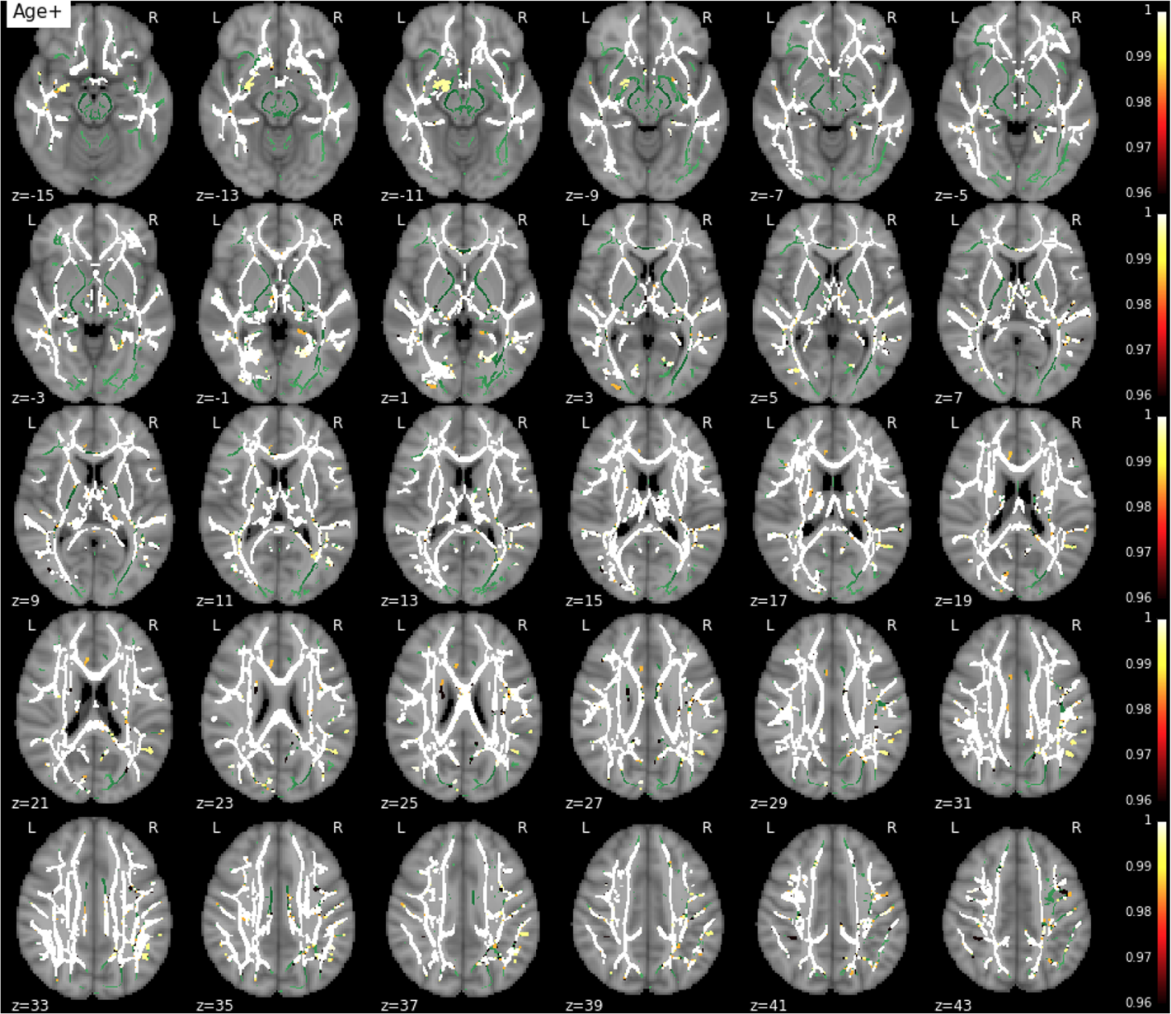
Effect of age on mean diffusivity. The white matter skeleton is shown in *green*. Suprathreshold clusters are presented in colors from *dark red* to *white* (1 − *p* > 0.95, familywise error rate- and threshold-free cluster enhancement-corrected)

**Fig. 7:**
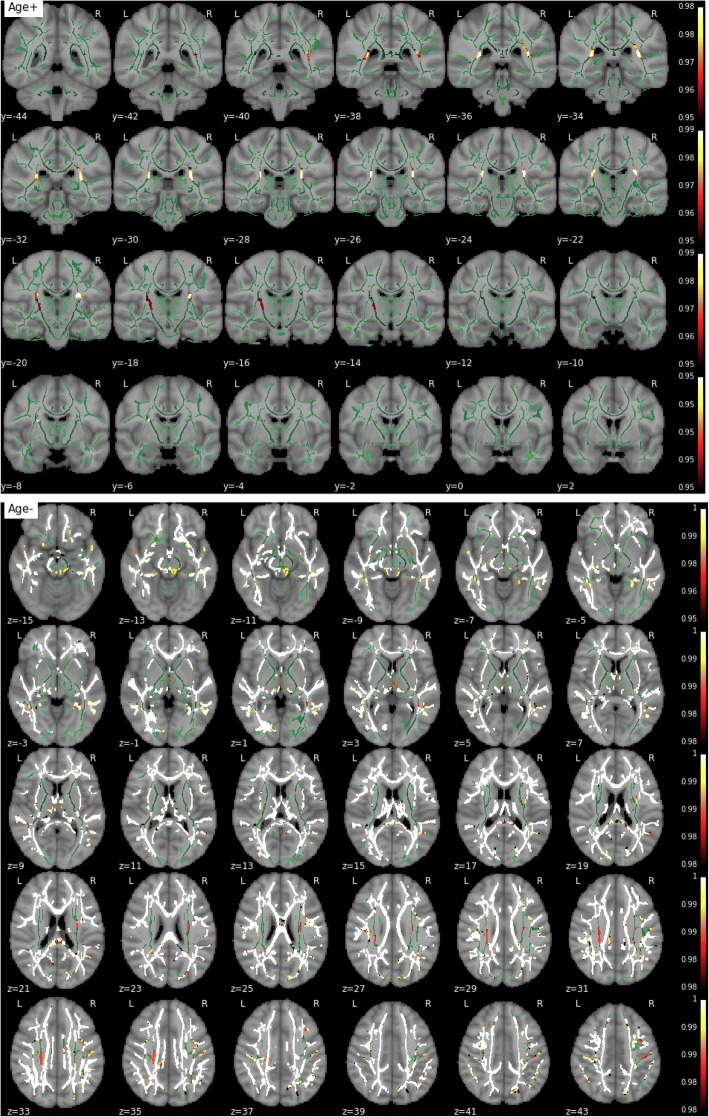
Effect of age on fractional anisotropy. The white matter skeleton is shown in *green*. Suprathreshold clusters are presented in colors from *dark red* to *white* (1 − *p* > 0.95, familywise error rate- and threshold-free cluster enhancement-corrected)

MD, RD, and AxD increased with age in the vast majority of the skeletonized WM, demonstrating previously well-documented results regarding age-related changes in diffusivity (Fig. 6). On the contrary, FA decreased with age in extensive regions but also showed a significant age-related increase in bilateral corticospinal tracts (Fig. 7). Educational attainment showed no significant effect on any of the diffusion parameters. No voxel demonstrated a significant association between any parametric map and global volumes of WM lesions or Fazekas scores in this dataset. Similarly, no global or regional effect of WMH load was detected in these respective complementary analyses.

### Matched sample analysis

In a similar fashion as in the initial full dataset, ε4 homozygotes showed increased values in MD, RD, and AxD as compared with other subjects. Consistent with our initial results, significant clusters appeared in regions such as SLF, IFOF, and forceps minor in decreasing order of spatial extent. Again, differences measured in RD were more extended than in AxD. ε4 Homozygotes also had significantly lower FA than other subjects in regions including forceps minor, bilateral IFOF, SLF, and anterior thalamic radiations. This difference was not observed initially in the original whole dataset. Contrast maps are presented in Additional file 1: Figures S1 to S4.

### Whole-brain voxel-wise analysis

*APOE-*ε4 homozygotes showed significantly increased MD as compared with other subjects. Significant clusters were found bilaterally in the WM essentially in SLF (Additional file 1: Figure S5). FA compared between genotype groups revealed no significant differences, in line with our results obtained using TBSS on the whole dataset.

## Discussion

The present study points to the existence of WM microstructural changes in cognitively normal adults carrying the ε4 allele. This effect was significant when we tested both the recessive and additive models. These results suggest that the ε4 allele adds extra burden to known age-related changes, especially for those individuals carrying two copies of the risk allele. In the brain, the *APOE* protein mediates neuronal delivery of cholesterol, which is an essential component for axonal growth, synaptic formation, and remodeling [2]. Because the ApoE-ε4 isoform of the protein is less efficient than ApoE-ε3 and ApoE-ε2 in transporting brain cholesterol [41], our findings could be interpreted as the result of a dysregulation in cholesterol homeostasis, which might contribute to the increased risk of AD observed in the ε4-homozygous group.

In support of this interpretation, it is worth noting that findings in RD and AxD show distinct patterns. Differences in RD but not in AxD are quite typically reported in AD risk studies [14, 16, 18, 42]. Considering that changes in AxD (in addition to RD increases and FA decreases) are observed in symptomatic AD, this would strengthen the idea that both correspond to distinct stages of WM degeneration. The smaller effects in AxD than in RD in healthy at-risk participants would suggest a disruption of the myelin sheath rather than pure axonal damage [42, 43]. A plausible explanation of this finding is that *APOE-*ε4 homozygotes, who lack expression of the more functional isoforms of the protein, have thinner myelin sheaths that what would correspond to their age. A thinner myelin sheath would decrease the electrical isolation of the axons, thus negatively influencing transmission speed [44] and demanding a higher metabolic consumption to sustain neurotransmission [45]. Such an effect would be in addition to the metabolic deficits associated to *APOE*-ε4 even in cognitively healthy populations [46, 47]. Therefore, increased metabolic demand coupled with an impaired neuroenergetic capability might explain the observed WM microstructural changes accelerating the effects of aging of WM microstructure in the *APOE*-ε4 homozygous group and would render this group more vulnerable to brain insults associated with AD. For instance, impaired cerebral metabolism could compromise the ability of microglia to remove amyloid deposition and might underlie the observed earlier and faster rate of amyloid accumulation in *APOE*-ε4 homozygotes [3].

The effects of *APOE* are essentially observed in regions known to be targets of AD pathology. Among them, SLF fibers showed the largest effect and are indeed known to be affected in AD [20, 24, 48–50] and in mild cognitive impairment [2, 22]. Effects appear stronger in posterior regions including the temporal and parietal lobes, whereas a number of studies demonstrated widespread effects, including frontal regions [12, 51]. Ryan et al. interpreted such widespread effects as a combination between ε4-related effects, which affect more posterior regions, and normal aging-related effects, which involve more frontal regions. On one hand, the younger age of the participants in the present study may partially explain the predominance of posterior changes because genotypic effects would predominate over age-related changes. On the other hand, the fact that posterior WM corresponds mainly to late-myelinating fiber pathways is in line with the retrogenesis model proposed by some authors [50, 52]. This model proposes that brain regions that are myelinated later in development tend to be more vulnerable to age-related damage. These regions include cortical association areas that recapitulate the spatial spread of AD lesions in reverse [53] and which support the cognitive functions that decline earlier in AD [54].

The differences partition into recessive and additive components. Recessive contrasts show larger clusters, but all significant voxels from recessive contrast maps include the ones from additive maps. Hence, the strongest differences that emerged in our dataset discriminate the homozygotes against other subjects. This stands out from a number of reported results about carriers differing from noncarriers [14, 18, 19], which would consequently suggest the existence of a rather dominant effect (with one or two copies of the risk allele). This difference may find explanation in the specific age ranges and distributions of the studied samples, most of which are older than in the present study. In such way, younger ε4 homozygotes in our study might be showing a behavior similar to older heterozygotes in other studies. In this regard, even though the genotypic effect observed in the present study does not interact with age, this same effect may become apparent in heterozygotes of older age. It is also worth noting that these studies opt for pooling ε4 carriers together, generally by lack of sufficient ε4 homozygotes, to be able to keep them as a distinct group. In the present study, the high number of homozygotes allowed us to study them as an individual group and to differentiate additive from recessive effects.

No significant differences in FA emerged between genotypic groups in our dataset. In contrast, researchers in a number of studies [12, 14–17, 19] have reported decreased anisotropy in cognitively healthy ε4 carriers. A possible explanation for this may again derive from the age ranges represented in these studies. Participants in our dataset, especially those at risk of developing AD, are at an early stage where FA is still not sensitive enough to measure WM alteration. Moreover, Acosta-Cabronero et al. [55] pointed out a possible scenario where absolute diffusivities increase with FA remaining stable because these parameters are mathematically related. Our results may be a plausible example of this scenario, and we hypothesize that when moving toward later stages, differences in FA, only emerging as stable trends in our data, will become significant. Interestingly, we observed possible evidence of this difference in FA when performing the same analysis on a smaller subsample using age-matched genotype groups, where homozygotes ε4 carriers revealed decreased anisotropy compared with other subjects.

Some groups of subjects revealed significant demographic differences in our dataset. In particular, homozygotes appeared to be significantly younger than others. Given the usual known direction of age-related changes on diffusion tensor imaging (DTI) parameters, the young age of these participants may give them an advantage by adding a “protective effect” (though assuming the absence of any pleiotropic expression of *APOE*) and may therefore hinder findings related to genotypic influence. Despite such heterogeneity in the dataset, these subjects still showed significant changes as compared with others. Previous researchers also investigated a potential protective effect attributed to the ε2 allele. To account for the potential influence of these two factors (age difference and ε2 allele), the same analytical protocol was run on a subsample consisting of three age-matched groups excluding ε2 carriers. As described in Additional file 1: Appendix A, results from this supplementary analysis were rigorously in line with those obtained with the original dataset. This confirms the earliness of the burden associated with *APOE*-ε4 homozygotes while discarding any influence from the ε2 allele. The number of ε2 carriers in our dataset remains far from being sufficient to allow us to assess the specific advantage attributed to this allele. Other modifiable risk factors may contribute to the changes measured in the WM microstructure. In particular, in this work, we evaluated the effect of educational attainment and found no significant voxel. Because pathological hyperintensities may also affect WM under the influence of cardiovascular and genotypic risk factors, associations between diffusion parameters and Fazekas scores or volumes of WM lesions were assessed and no association emerged. The explanation for this lack of influence may lie in the low burden of WM hyperintensities found in our sample.

Although having been studied in the WM for the most part, diffusion changes also take place in the GM and have been described in recent papers [56, 57]. Although there is some evidence that microstructural changes come early in the pathological cascade, whether the earliest changes occur in cortical regions or in WM fiber fascicles is still under debate. This present study describes differences observed using a tract-based analysis technique. Although some methodological warnings have been raised in relation to using this technique, as described previously [58, 59], and although the technique may not be used for areas other than WM, it generally provides increased sensitivity in the detection of changes along the most stable fiber tracks and has been widely used as such in many previous works reported in the literature. Nevertheless, we performed a complementary whole-brain voxel-based (SPM) analysis using the same statistical models, contrasts, and dataset (–Additional file 1: Appendix B). The resulting significant clusters were exclusively located in the WM without using any prior anatomical assumptions in the detection. This would support the hypothesis that microstructural changes occur exclusively in the WM. However, because previous studies have shown a nonmonotonous behavior of cortical water diffusivity with progressive preclinical AD stages [56], we cannot rule out the presence of significant effects on GM. Unfortunately, the lack of core AD biomarkers in this study prevented us from testing this hypothesis. Besides, it could be argued that voxel-based analyses may suffer from the inclusion of signal from cerebrospinal fluid in GM voxels, which would reduce sensitivity within cortical regions. To overcome this, one could then consider opting for different methods, such as using surface-based schemes [56], which would avoid smoothing using a 3D kernel.

Samples of “healthy” participants are of high interest because they allow study of structural markers at an early stage before deviating from the course of normal aging. However, such studies are often limited by the lack of additional markers that would discriminate preclinical subjects at the earliest stages of AD, such as cerebrospinal fluid markers and brain amyloid burden. Thus, such studies, including this present one, face the risk of having individuals with preclinical AD overrepresented within their *APOE*-ε4 groups. To mitigate this, we will have access to follow-up information that will allow us to better stratify our sample with respect to preclinical AD research criteria [60] and to minimize the risk of including persons with subtle cognitive decline in the healthy group. In particular, a fraction of this cohort will undergo complementary examination including positron emission tomographic imaging and lumbar puncture. (*See* [34] for a detailed description of the various arms of the study.) To date, very few studies have included both structural metrics (e.g., DTI and indices of AD pathology such as cerebrospinal fluid and brain amyloid markers at the preclinical stage). The screening of these individuals will then allow the link between microstructure and cognition to be investigated and compared between healthy and preclinical subjects. A further reason for including study subjects prior to development of disease would be to assess how baseline diffusivity parameters may predict the time before clinical onset, considering especially that *APOE*-ε4 is known to have a lower age at onset, in a gene-dose-dependent manner.

The major strength of our study lies in having recruited a relatively young, cognitively healthy sample, with a very large number of *APOE-*ε4 homozygotes. This allowed us to study individuals at three levels of risk, thus building on most published studies that compared carriers vs noncarriers. Our findings are robust, as confirmed by several methodological approaches, and are not driven by cerebrovascular disease, as confirmed by ruling out any impact of WMH. However, there are some limitations to our work. The most notable one is that we do not know the amyloid status of the studied participants. Cognitively healthy *APOE-*ε4 homozygotes have been reported to show a significantly higher prevalence of cerebral amyloid pathology. At the mean age of our homozygote group (55 years), approximately 50% of these individuals display abnormal levels of amyloid biomarkers, as compared with only 10% of noncarriers and about 20% of carriers of a single ε4 allele [61]. However, the lack of interaction with age in our findings, in agreement with some previous reports [14, 18], is supportive of our findings not being driven by amyloid status. The fact that no inflection point in the association between RD was found around this age supports the hypothesis that the thinning of the myelin sheath is a genetically determined trait in these subjects rather than a downstream effect of amyloid deposition. dMRI studies in *APOE-*ε4 homozygote children and adolescents are needed to confirm this hypothesis. In middle-aged populations, amyloid biomarkers and longitudinal data would obviously be necessary to discern the influence of amyloid deposition in dMRI scalars, and actually, some previous works suggest that microstructural properties in the WM are subject to the combined influence of age and genotype [12, 20].

## Conclusions

Our results confirm that carrying the *APOE*-ε4 allele confers an additional burden to the normal age-related changes observed in WM in cognitively healthy individuals. This burden emerges as differential changes in dMRI parameters, essentially in diffusivity, suggesting early affection of the fibers of the myelin sheath at a stage predating axonal loss and typically resulting in decreases of anisotropy. With the uniquely high number of homozygotes in our dataset, our study shows that carrying two copies of the ε4 allele is also associated with a significantly higher impact on the WM microstructure.

## Additional file

Additional file 1: Supplementary data. (DOCX 1884 kb)

## Abbreviations

AD: Alzheimer’s disease
ADC: Apparent diffusion coefficient
*APOE*: Apolipoprotein E gene
AxD: Axial diffusivity
dMRI: Diffusion magnetic resonance imaging
DTI: Diffusion tensor imaging
DW: Diffusion-weighted
FA: Fractional anisotropy
FH: Family history
GM: Gray matter
IFOF: Inferior fronto-occipital fasciculus
ILF: Inferior longitudinal fasciculi
LOAD: Late-onset Alzheimer’s disease
MD: Mean diffusivity
MRI: Magnetic resonance imaging
RD: Radial diffusivity
SLF: Superior longitudinal fasciculi
SPM: Statistical Parametric Mapping
TBSS: Tract-based spatial statistics
VBM: Voxel-based morphometry
WM: White matter
WMH: White matter hyperintensities
