# Supplementary material for: White matter microstructure is altered in cognitively normal middle-aged APOE-ε4 homozygotes
Source: Alzheimers Res Ther. 2018 May 24;10:48. doi: 10.1186/s13195-018-0375-x (PMC5968505; doi:10.1186/s13195-018-0375-x)
Supplement: Supplementary file 1 — Supplementary data. (DOCX 1884 kb) [file 13195_2018_375_MOESM1_ESM.docx]

**Additional file 1**

**Appendix A: Matched sample analysis**

**Methods**

From the 532 subjects of the initial dataset, an age-matched subsample was selected. This sample consisted of 3 groups of 65 ε4 homozygotes, 65 ε4 heterozygotes and 65 non-carriers.

**Table S1 – Sample characteristics**

|  | **Total sample**  **(N = 195)** | | **NC**  **(N = 65)** | | **HE**  **(N = 65)** | | **HO**  **(N = 65)** | | **Inferential**  **statistics** |
| --- | --- | --- | --- | --- | --- | --- | --- | --- | --- |
|  | *M* | *SD* | *M* | *SD* | *M* | *SD* | *M* | *SD* |  |
| **Age*** | 54.73 | 6.19 | 54.75 | 6.20 | 54.74 | 6.23 | 54.75 | 6.21 | F = 0.00; P = 0.99 |
| **Education*** | 13.53 | 3.50 | 13.58 | 3.50 | 13.63 | 3.60 | 13.58 | 3.50 | F = 0.09; P = 0.91 |
| **MMSE** | 29.16 | 0.98 | 29.17 | 1.02 | 29.08 | 1.14 | 29.17 | 1.02 | F = 0.56; P = 0.57 |
| **TFR** | 17.28 | 5.17 | 16.91 | 5.44 | 17.22 | 4.94 | 16.91 | 5.44 | F = 0.42; P = 0.66 |
| **TPR** | 24.66 | 4.16 | 24.18 | 4.17 | 24.63 | 4.16 | 24.18 | 4.57 | F = 0.94; P = 0.39 |
| **Male/female** | 74/121 | | 19/46 | | 31/34 | | 24/41 | | χ^2^ = 4.75; P = 0.09 |

NC = Non-carriers; HE = *ε4*-Heterozygous; HO = *ε4*-Homozygous; MMSE = Mini-Mental State Examination score; TPR = Total Paired Recall; TFR = Total Free Recall; M = mean; SD = standard deviation

*Indicated in years

The exact same analysis protocol was carried out on this matched sample using TBSS. The same statistical models were implemented and the same contrasts were estimated (2.5).

**Results**

Contrasts maps are presented in Figures S1 to S4.

In a similar fashion as with using the initial full dataset, ε4 homozygotes show increased values in MD, RD and AxD as compared to other subjects. Consistently with our initial results, significant clusters appear in regions such as SLF, IFOF and forceps minor. Again, differences measured in RD are more extended than in AxD. ε4 homozygotes also reveal a significantly lower FA as compared to other subjects in regions including - by decreasing order of spatial extent - forceps minor, bilateral IFOF, SLF and anterior thalamic radiations. This difference was not observed initially in the original full dataset.


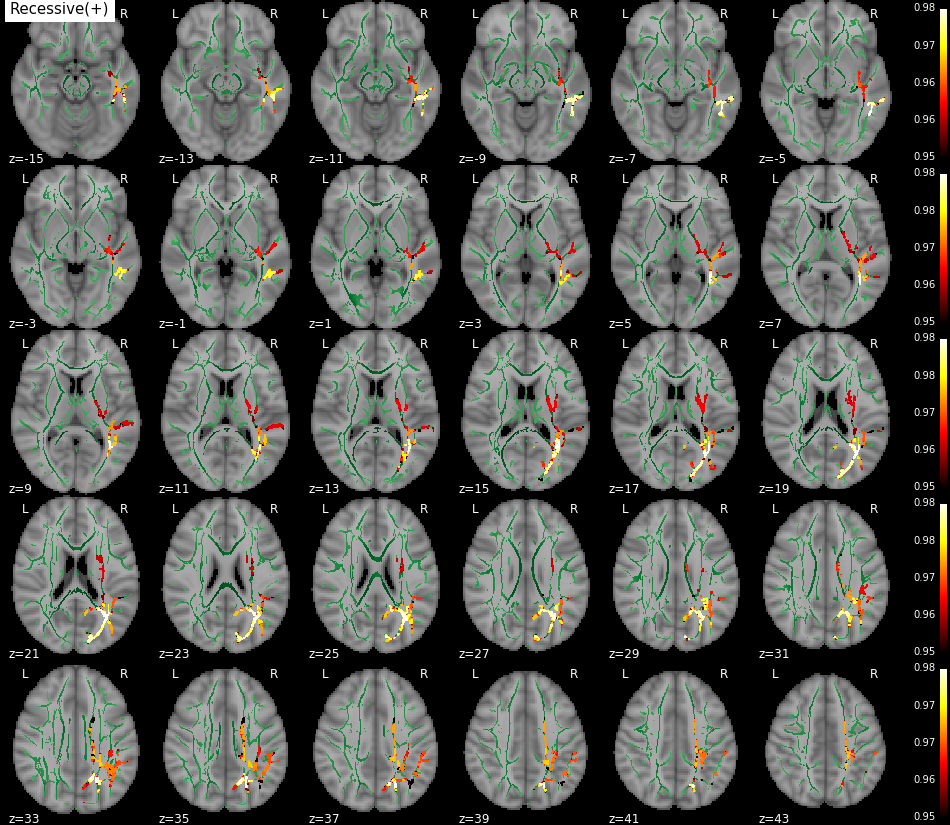


**Figure S1** - Effect of *APOE* on MD on an age-matched subsample - only a recessive effect was observed - only contrast maps associated with higher MD in ε4 carriers showed significant voxels - the white matter skeleton is shown in green - supra-threshold clusters are presented in colors from dark red to white (1-p>0.95 FWE-TFCE corrected)


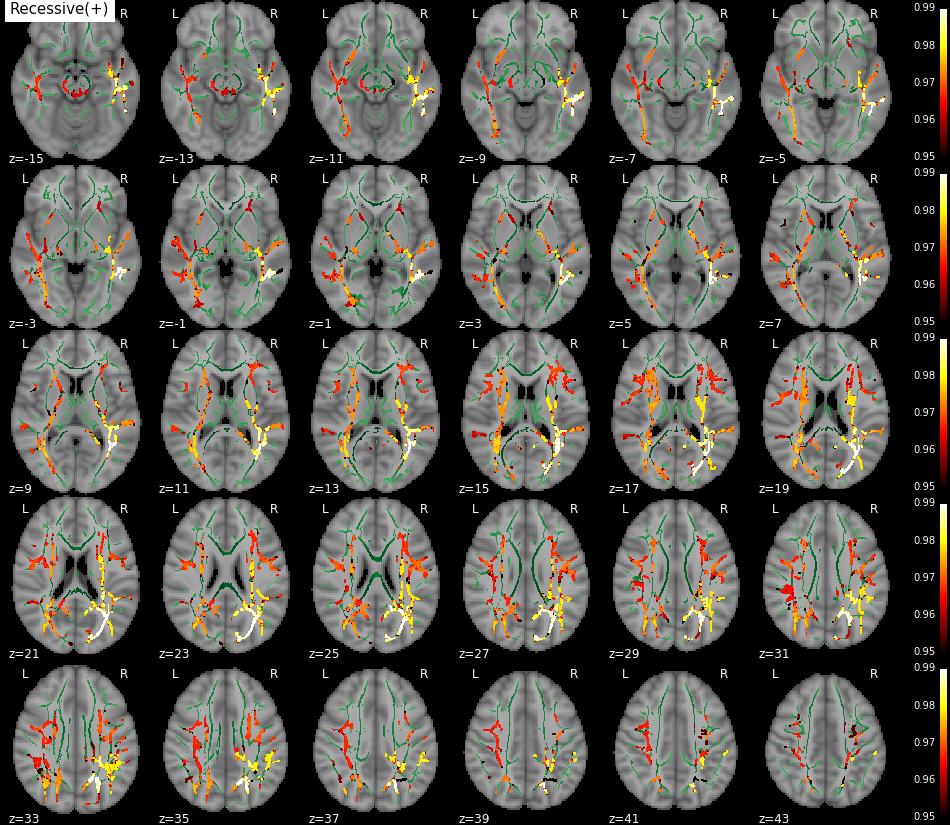


**Figure S2** - Effect of *APOE* on RD on an age-matched subsample - only a recessive effect was observed - only contrast maps associated with higher RD in ε4 carriers showed significant voxels - the white matter skeleton is shown in green - supra-threshold clusters are presented in colors from dark red to white (1-p>0.95 FWE-TFCE corrected)


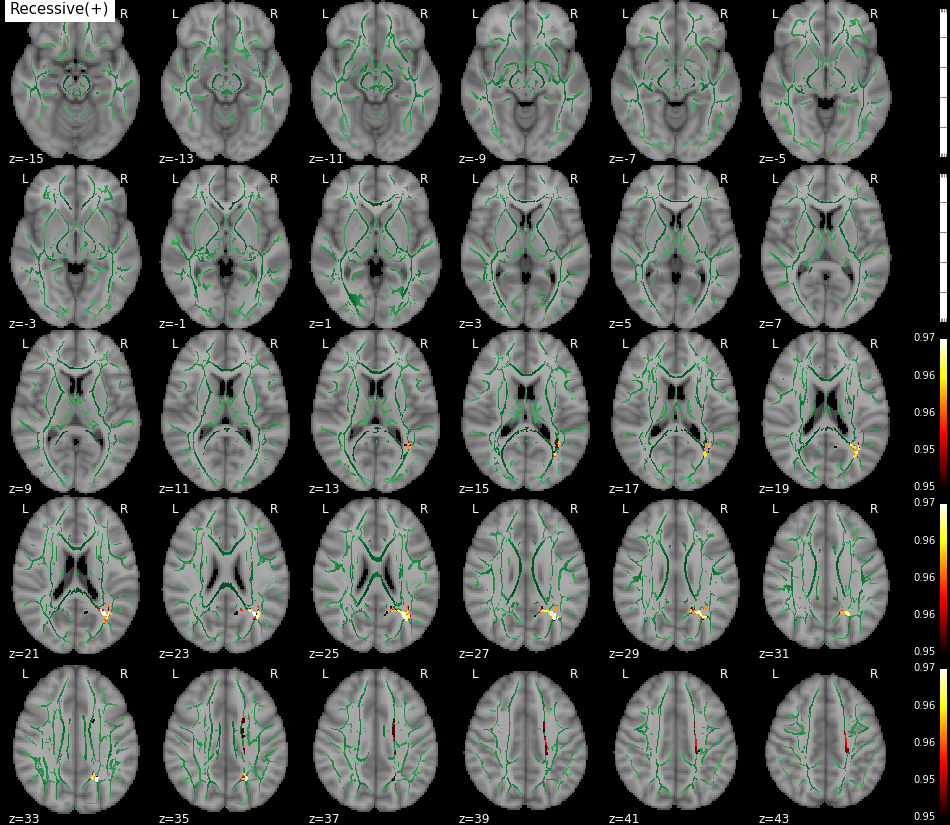


**Figure S3** - Effect of *APOE* on AxD on an age-matched subsample - only a recessive effect was observed - only contrast maps associated with higher AxD in ε4 carriers showed significant voxels - the white matter skeleton is shown in green - supra-threshold clusters are presented in colors from dark red to white (1-p>0.95 FWE-TFCE corrected)


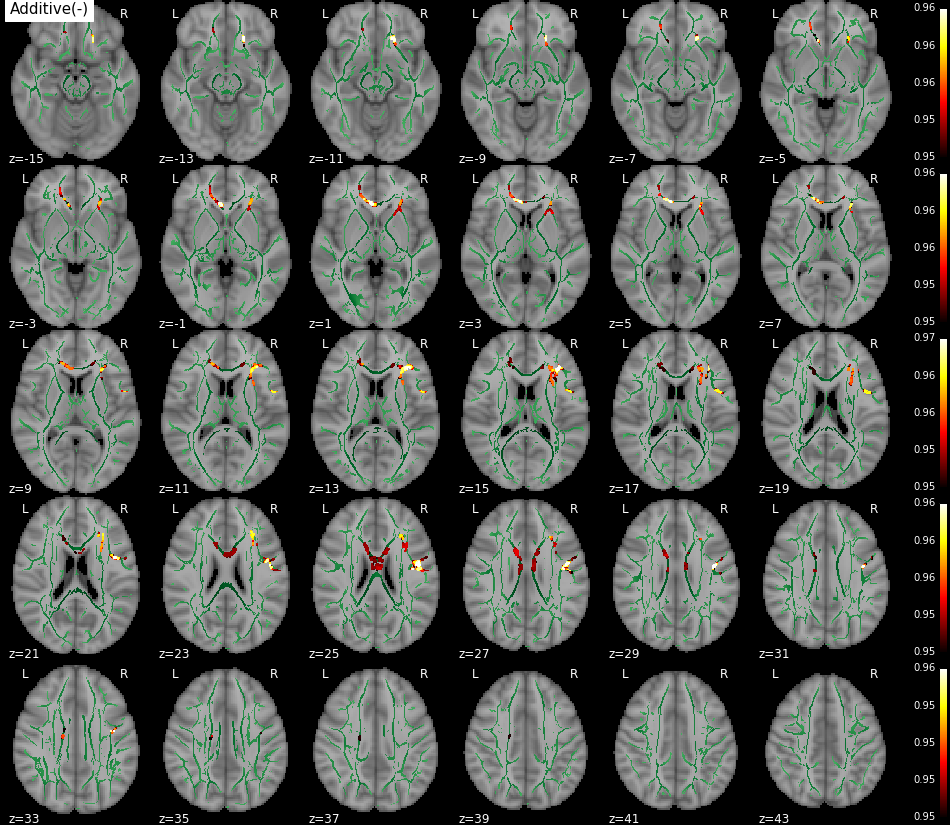


**Figure S4** - Effect of *APOE* on FA on an age-matched subsample - only a recessive effect was observed - only contrast maps associated with lower FA in ε4 carriers showed significant voxels - the white matter skeleton is shown in green - supra-threshold clusters are presented in colors from dark red to white (1-p>0.95 FWE-TFCE corrected)

**Appendix B: Voxel-wise whole brain exploratory analysis**

**Image data acquisition**

Along with DW images (2.3), structural 3D high-resolution T1-weighted images were collected on the same single standard 3T General Electric scanner (GE Discovery MR750 W) using a fast spoiled gradient-echo (FSPGR) sequence with the following parameters: voxel size = 1mm3 isotropic, Repetition Time [TR] = 6.16 ms, Echo Time [TE] = 2.33 ms, inversion time [TI] = 450 ms, matrix size = 256 x 256 x 174, flip angle = 12º.

**Image processing**

A first spatial normalization was applied to T1 images using DARTEL [[1](#_ENREF_1)] to generate an anatomical template. DW images (MD and FA maps) were first registered to T1 images, then preprocessed by masking out every voxel associated to a probability of belonging to cerebrospinal fluid (CSF) higher than 0.5 - this in order to mitigate possible contamination from CSF signal. Preprocessed images were finally warped to the reference template applying the transformations returned by DARTEL. We implemented the same models as described in section 2.5 in a voxel-wise multiple regression analysis. From the 532 subjects with DWI data used in the TBSS analysis, a total of 32 had to be discarded because of poor quality of their T1 image or unsuccessful normalization. 500 subjects were therefore included in this analysis.

**Statistical analysis**

We used SPM12 to run a multiple linear regression analysis. We employed the same statistical models as in 2.5 and estimated the same contrasts.

**Results**

FA compared between genotypic groups revealed no significant differences, in a similar fashion as in TBSS. *APOE* ε4 homozygotes showed significantly increased MD as compared to other subjects. Significant clusters are essentially found in the WM in bilateral SLF (Figure S5).

**
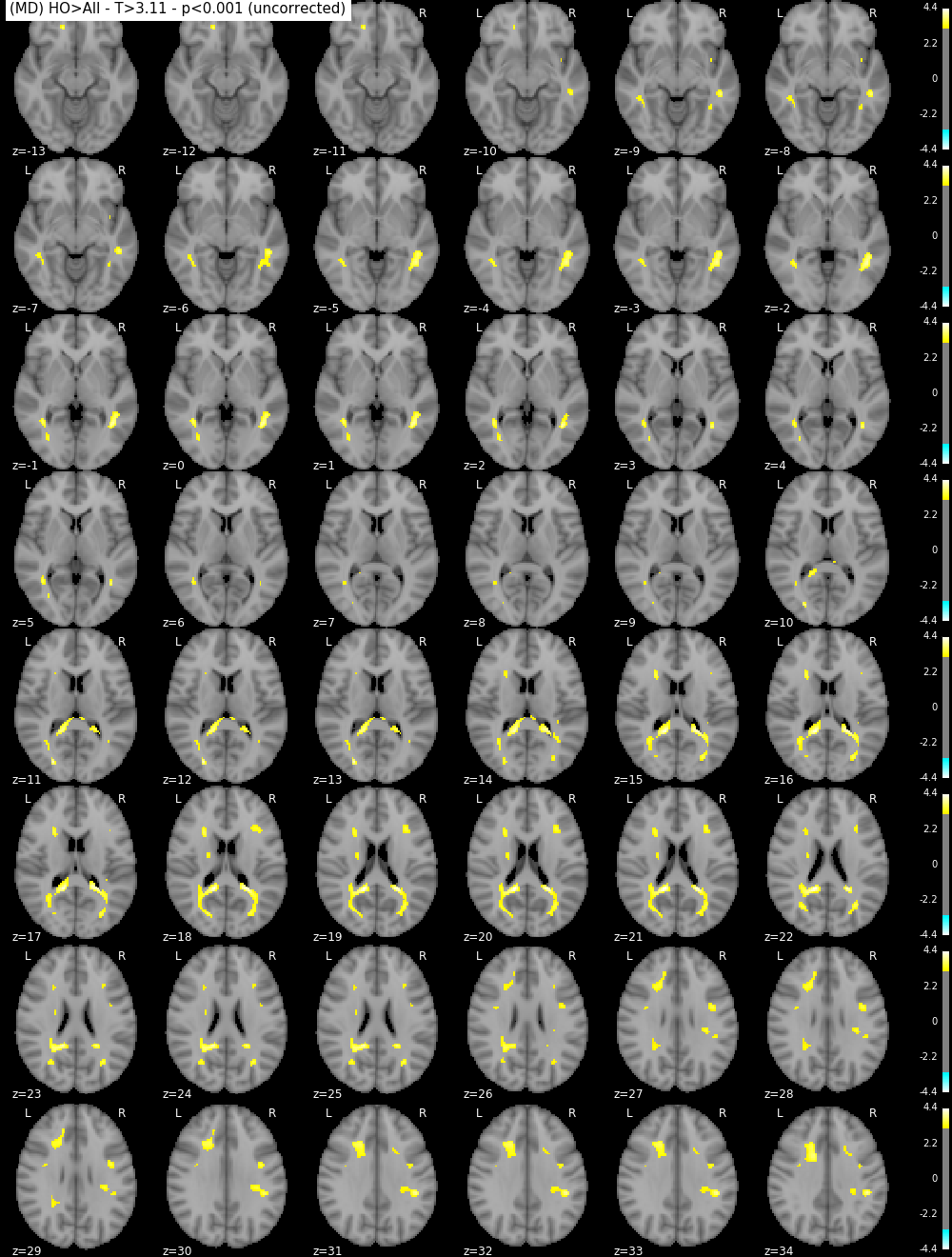
**

**Figure S5** **-** Effect of *APOE* on MD using whole-brain voxel-wise analysis - only a recessive effect was observed - only contrast maps associated with higher MD in ε4 carriers showed significant voxels (uncorrected - p<.001)

**References**

1. Ashburner, J., *A fast diffeomorphic image registration algorithm.* Neuroimage, 2007. 38(1): p. 95-113.
